# Upper Tract Urothelial Carcinoma in the Genetically Predisposed Patient: Role of Urinary Markers in Predicting Recurrence

**DOI:** 10.1089/cren.2016.0124

**Published:** 2016-12-01

**Authors:** Wei Phin Tan, Nahom Tecle, Patrick Whelan, Andrea Strong, Leslie A. Deane

**Affiliations:** Department of Urology, Rush University Medical Center, Chicago, Illinois.

**Keywords:** Cxbladder, urine cytology, FISH assay, urothelial cancer

## Abstract

***Background:*** Upper tract urothelial carcinoma (UTUC) is an uncommon disease that is diagnosed clinically by the selective use of urine cytology, urine biomarkers, and imaging of the upper tract. We present a case of a patient with Lynch syndrome and high-grade UTUC that was diagnosed by an abnormal Cxbladder assay, prompting further endoscopic examination.

***Case Presentation:*** A 59-year-old Caucasian female with a history of endometrial cancer and bladder cancer with Lynch syndrome presented for evaluation of recurrent urothelial carcinoma. Her previous bladder tumors have been T1 high grade and Ta high grade and have been treated with resection and multiple cycles of intravesical Bacillus Calmette–Guerin (BCG) therapy. She had also undergone a robotic left distal ureterectomy and psoas hitch for a high-grade distal ureteral tumor. Surveillance cystoscopy 7 months after revealed a biopsy-confirmed bladder tumor, which was resected, and she was started on maintenance BCG therapy. At presentation, follow-up urine cytology and UroVysion studies were negative. Cxbladder test was also initially negative. However, during close clinical monitoring, the Cxbladder test became positive. Cystoscopy was once more performed, which was unremarkable. Bilateral ureteroscopy was performed, revealing high-grade upper tract renal papillary carcinoma (UTUC) in the left renal pelvis. The patient declined a nephroureterectomy. She was treated with two sessions of holmium laser ablation of the left renal pelvis tumor and underwent 6 weekly courses of BCG + interferon instilled into her left renal pelvis using a 5F open-ended catheter. Repeat urine cytology, UroVysion, and Cxbladder tests were negative after completion of upper tract BCG therapy.

***Conclusion:*** Cxbladder test may be useful and an adjunct to urine cytology and the UroVysion FISH assay to evaluate patients at high risk for recurrent UTUC.

## Introduction

Upper tract urothelial carcinoma (UTUC) is a rare diagnosis that only accounts for 5% of urothelial carcinoma (UC).^1^ UTUC carries a poor prognosis and can have aggressive characteristics.^1^ The clinical diagnosis of UTUC is typically with the selective use of urinary cytology, urinary biomarkers, and imaging of the upper tract with particular focus on the collecting system. We describe a case in which recurrent UTUC was diagnosed using the Cxbladder urinary biomarker (Dunedin, New Zealand), while testing negative on urine cytology and UroVysion FISH assay (Lake Bluff, IL, USA) and before any imaging changes were visible on CT and MRI of the abdomen.

## Case Presentation

**FIG. 1.**
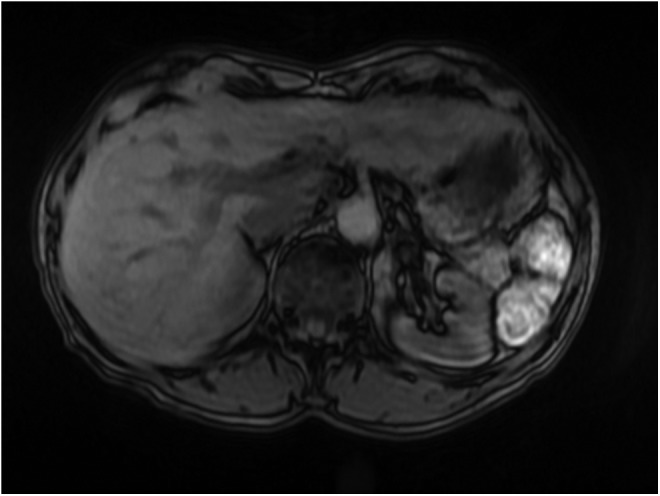
MRI of the abdomen showing normal appearance of the left renal pelvis in which high-grade transitional cell tumor was discovered on ureteroscopy.

**FIG. 2.**
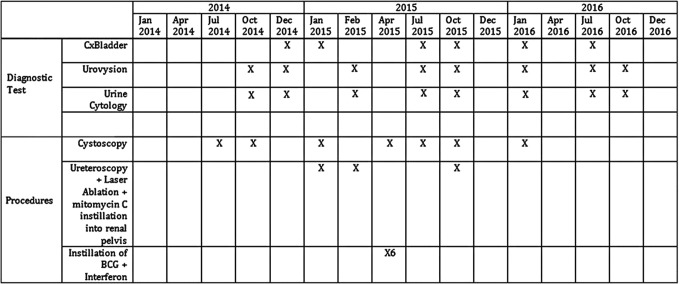
Timeline of urine biomarkers.

A 59-year-old female with a medical history of Lynch syndrome was seen at our center for ongoing surveillance. She previously had a robot-assisted left distal ureterectomy for high-grade tumor and had multiple transurethral resections for high-grade nonmuscle invasive bladder cancer. She had previously undergone two courses of induction Bacillus Calmette–Guerin (BCG) therapy for bladder cancer. On presentation, urine cytology, UroVysion FISH test, Cxbladder test, and surveillance cystoscopy were negative. During close clinical follow up, the Cxbladder test showed that the gene expression started to rise into the high range, whereas other urinary markers remained negative. No lesion could be identified in the collecting system on CT or MRI of the abdomen and pelvis (Fig. 1). This prompted bilateral diagnostic ureteroscopy using the Narrow Band Imaging settings of an URF-V2 digital ureteroscope by Olympus (Shinjuku, Tokyo, Japan). This revealed UTUC of the left renal pelvis and upper calix, which was biopsied and treated with holmium laser ablation, as the patient refused nephroureterectomy. After two sessions of laser ablation of the left renal pelvis and upper caliceal tumor, she was endoscopically tumor free. At the time of laser ablation, Mitomycin C was instilled into the upper tract through a ureteral catheter. Subsequently, she underwent induction BCG + interferon (6-week course) to the left renal pelvis through placement of a 5F open-ended catheter. One vial of BCG + interferon (1–8 × 10^8^ colony-forming unit/50 mm^3^ saline and 50 million units of interferon alpha 2b) was instilled into the left renal pelvis over 90 minutes each session. Follow-up cystoscopy, cytology, UroVysion, and Cxbladder tests were negative after treatment of the left UTUC. Nine months later, Cxbladder test once again showed increasing gene expression and repeat ureteroscopy showed a small recurrence in the upper calix, which was ablated. Cytology and FISH tests remained negative throughout. Figure 2 reveals the timeline of her clinical course.

## Discussion

There are multiple US Food and Drug Administration-approved biomarkers used to detect bladder cancer: nuclear matrix protein 22, qualitative bladder tumor antigen, and FISH.^1,2^

Cxbladder is a commercially available urine biomarker consisting of five biomarkers (MDK, HOXA13, CDC2, IGFBP5, and CXCR2) that extract messenger RNA from the urine to undergo a reverse transcription quantitative polymerase chain reaction, which converts RNA to DNA. The DNA is then amplified and detected based on the proportion of copies of abnormal DNA present. Each of the five biomarkers is quantified by a different probe and the relationship between the individual biomarkers is determined by a mathematical equation. The calculated outcome provides a measure of the probability of the presence of UC. The detection rate of the combination of CDC2, MDK, IGFBP5, and HOXA13 for stages Ta, T1, and >T1 urothelial cell carcinoma is 48%, 90%, and 100%, respectively, at a specificity of 85%. The combination of CDC2 and HOXA13 distinguished between grades 1 to 2 urothelial cell cancer and grade 3 or stage ≥T1 urothelial cell carcinoma with 80% specificity and sensitivity.^3^

The UroVysion FISH assay tests for abnormalities in chromosomes 3, 7, 9, and 17. UroVysion has been shown to have a high specificity and negative predictive value in detecting bladder cancer.^4^ In our patient, UroVysion and urine cytology were consistently negative. Cxbladder test showed the only abnormal finding, which prompted further endoscopic examination. Our patient refused a nephroureterectomy and was treated with two sessions of holmium laser ablation of UTUC in the left kidney. She then underwent induction BCG + interferon for 6 weeks, instilled through a 5F open-ended catheter over 90 minutes. She underwent a third ablation at the time of recurrence 9 months later. She was also subsequently started on three weekly maintenance BCG therapies. After completion of BCG therapy, urine cytology, UroVysion FISH test, and Cxbladder test all remained negative on 12 months follow-up.

## Conclusion

Cxbladder test may be useful and an adjunct to urine cytology and the UroVysion FISH assay to evaluate patients at high risk for recurrent UTUC. This case report adds to the emerging evidence that Cxbladder is a useful tool for monitoring the recurrence of UTUC.

## Abbreviations Used

UTUC: upper tract urothelial carcinoma
BCG: Bacillus Calmette–Guerin
